# Ultrasound Stimulations Induce Prolonged Depolarization and Fast Action Potentials in Leech Neurons

**DOI:** 10.1109/OJEMB.2019.2963474

**Published:** 2020-02-14

**Authors:** Francesca Dedola, Francesco Paolo Ulloa Severino, Nicolò Meneghetti, Théo Lemaire, Andrea Cafarelli, Leonardo Ricotti, Arianna Menciassi, Annarita Cutrone, Alberto Mazzoni, Silvestro Micera

**Affiliations:** ^1^ The Biorobotics InstituteScuola Superiore Sant'Anna 19005 Pisa 56025 Italy; Department of Excellence in Robotics and AIScuola Superiore Sant'Anna 19040 Pisa 56025 Italy; ^2^ Neuroscience AreaInternational School for Advanced Studies 27218 Trietse 341356 Italy; ^3^ Bertarelli Foundation Chair in Translational NeuroEngineering, Center for Neuroprosthetics and Institute of BioengineeringSchool of Engineering, Ecole Polytechnique Federale de Lausanne Lausanne 1015 Switzerland; ^4^ BioRobotics InstituteScuola Superiore Sant'Anna 19005 Pisa 56025 Italy; Department of Excellence in Robotics and AIScuola Superiore Sant'Anna Pisa 56025 Italy; Bertarelli Foundation Chair in Translational NeuroEngineering, Center for Neuroprosthetics and Institute of BioengineeringSchool of Engineering, Ecole Polytechnique Federale de Lausanne Lausanne 1015 Switzerland

**Keywords:** Action Potential, Depolarization, Leech, Neuromodulation, Ultrasounds

## Abstract

*Objective:* Ultrasound (US) stimulation carries the promise of a selective, reversible, and non-invasive modulation of neural activity without the need for genetic manipulation of neural structures. However, the mechanisms of US-induced generation of action potentials (APs) are still unclear. *Methods:* Here we address this issue by analyzing intracellularly recorded responses of leech nociceptive neurons to controlled delivery of US. *Results:* US induced a depolarization linearly accumulating in time and outlasting the duration of the stimulation. Spiking activity was reliably induced for an optimal US intensity range. Moreover, we found that APs induced by US differ in smaller amplitude and faster repolarization from those induced by electrical stimulation in the same cell but display the same repolarization rate. *Conclusions:* These results shed light on the mechanism by which spikes are induced by US and pave the way for designing more efficient US stimulation patterns.

## I. Introduction

Ultrasound (US) stimulation is an efficient mean to interact non-invasively with the human body. Beyond its conventional use as an imaging modality [1], US is now also applied for therapeutic interventions such as ablation therapies and blood-brain barrier reversible opening for drug delivery [2]. Such applications rely on the specific nature of acoustic waves that can be accurately steered through biological tissue, offering the ability to concentrate acoustic energy within small volumes (∼mm^3^) around deep anatomical targets [3].

Low-intensity low-frequency ultrasound (LILFU) can elicit APs in ex-vivo mouse brain and hippocampal slice cultures [4]. US neuromodulatory effects have been recently studied on various neural targets [5] with a clear translation path from ex-vivo preparations [4], [6], to animal models [7]–[15] including non-human primates [16]–[18], and human subjects [19]–[24]. However, while the ability of US to modulate neural activity has been extensively confirmed, discrepancies remain about the exact influence of each stimulation parameter (carrier frequency f_c_, peak pressure amplitude, stimulation duration, pulse-repetition frequency and duty cycle (DC)) on neural activity, and behavioral responses. Hence, despite a decade of intense investigation, the underlying mechanism of action by which US triggers neuronal excitation/inhibition is still unclear and a predictive model relating the effects on neurons with the US parameters is missing.

Here, in order to tackle these issues, we investigated neural responses to controlled US in a simple nervous system: the medicinal leech (*Hirudo medicinalis*). This invertebrate model can generate a broad range of behaviors [25], [26], but possesses a simple nervous system that can be easily accessed to extract individual ganglia, the anatomical organization of which is highly conserved across specimens [27]. Neurons within a ganglion are functionally characterized and identifiable by their position, size, and electrophysiological profile [28], [29], and offer very reliable responses to EL stimulation. Finally, it is possible to record viable neural responses from an isolated ganglion for extended periods of time [29].

Using this animal model, we systematically explored the US parameter space and recorded direct individual neural responses in isolated leech ganglia, in a controlled environment where the number and influence of external factors was kept to a minimum. We aimed at identifying relevant acoustic parameters governing neural responses, evaluating excitation thresholds, and comparing obtained spike waveforms with that of EL evoked spikes. We focused our analysis on nociceptive (N) mechanosensory cells that exhibit a robust and well-characterized response to EL stimulation, and whose characteristics are predominantly due to the abundance on the membrane of Na^+^ channels, which have been recently indicated to be influenced by US [13].

## II. Results

**Figure 1.**
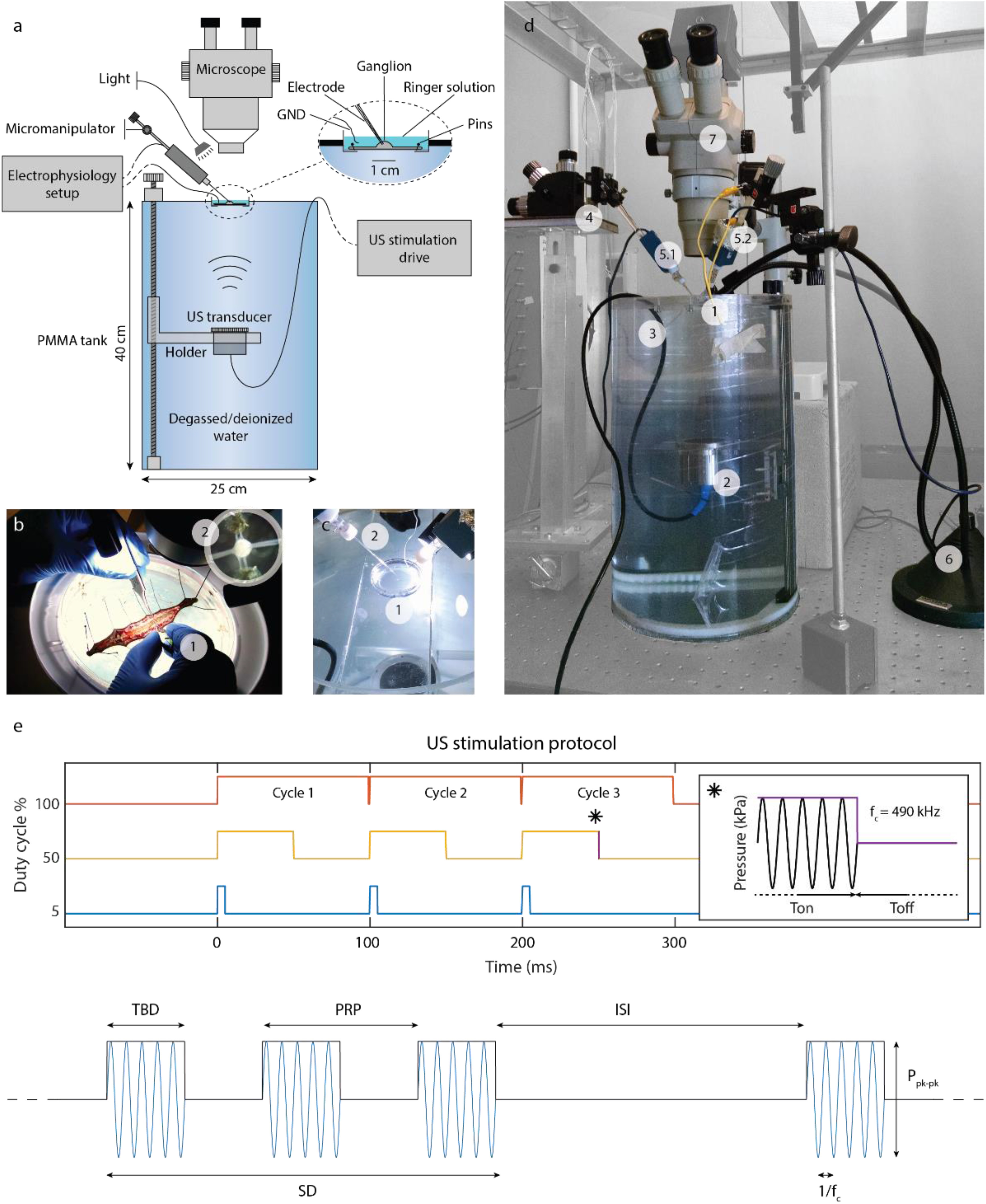
Experimental setup. (a) Illustrative scheme of the setup. (b) Leech dissection and ganglia chain exposition (1). On the top right corner, a detail of the extracted leech ganglion pinned onto the PDMS substrate (2). (c) Focus on the Petri dish with a pinned ganglion (1) and the glass capillary containing the Ag/AgCl electrode for intracellular recording (2). (d) The Petri dish with the pinned ganglion was positioned on top of the experimental setup (1). The setup included a US transducer (2) immersed in a tank (3) full of degassed deionized water used for US, and an electrophysiology setup. A micromanipulator (4) allows for fine positioning of the electrodes on ganglion surface. In this configuration two electrodes (5.1–5.2) are connected to the electrophysiology setup for recording. A light source (6) and an optical microscope (7) are used for cell identification and impalement. (e) Temporal protocol of US: DC 5% (blue), T-on is 5 ms, T-off 95 ms; DC 50% (yellow), T-on 50 ms, T-off 50 ms; DC 100% (brown), T-on 100 ms. The tone burst duration is equal to T-on in case of pulsed stimulation and the pulse repetition period (PRP) is 100 ms; the stimulation duration (SD) is of 300 ms for the 3 temporal protocols. Each stimulation (SD 300 ms) is repeated 3 times during a recording session whit an inter stimulation interval (ISI) of 20–30 s. In the inset, temporal evolution of US (violet); transducer central frequency is 490 kHz.

We developed a setup (Fig. 1(a)–(d)) to stimulate cells with ultrasound from isolated leech ganglia (Fig. 1(b)–(c)). We analyzed responses of N mechanosensory neurons to US (see Materials and Methods and Fig. 1(e) for details) comparing them to the responses to EL stimulation. We first analyzed US-induced subthreshold depolarization, then US-induced firing activity, and finally US-induced AP characteristics were compared with those of EL triggered spikes.

### A. Subthreshold Responses to US Stimulation

**Figure 2.**
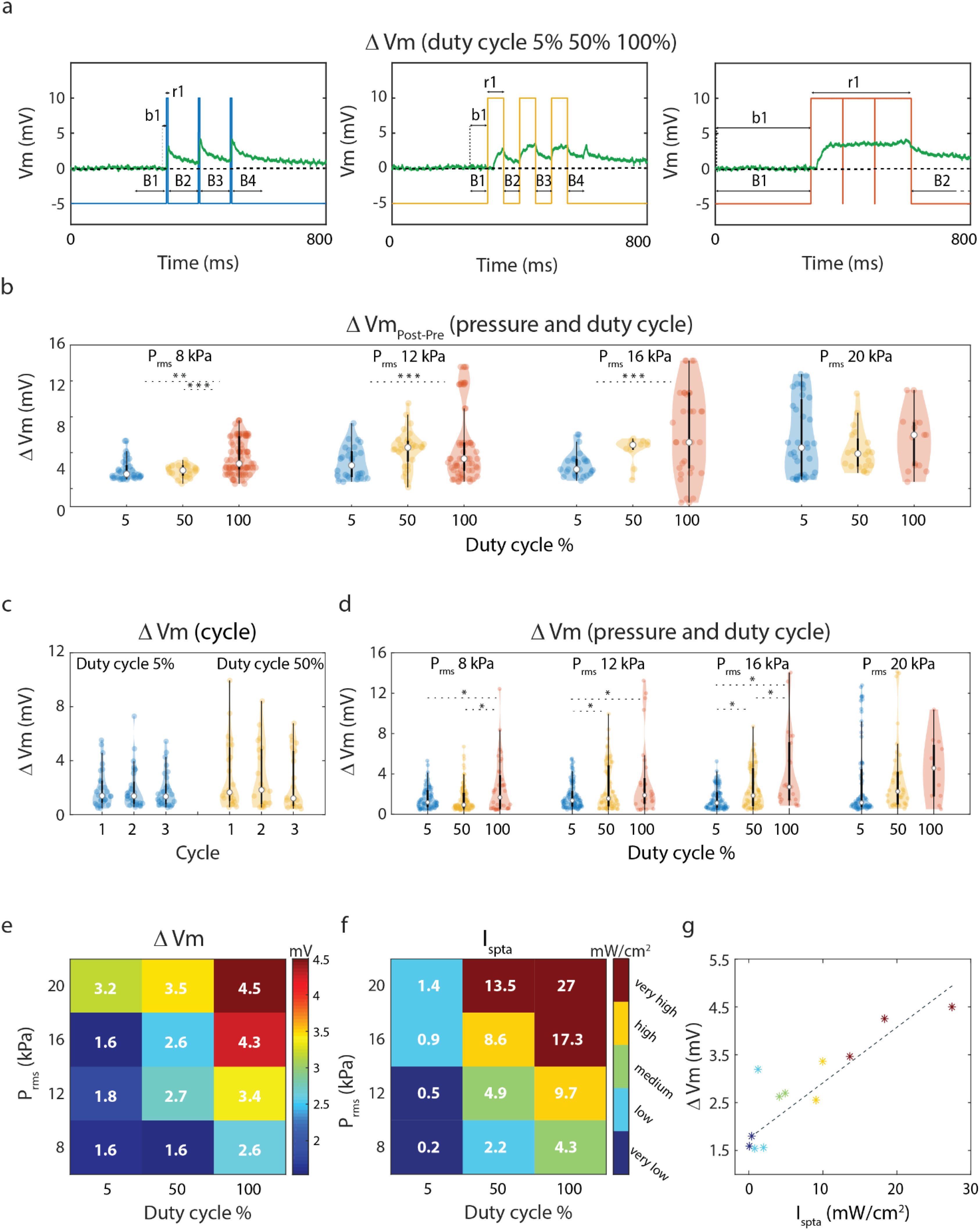
US parameters and membrane depolarization. (a) Definition of response and baseline interval for each DC; in case of 100% DC, baseline and response time interval is 300 ms; in case of 5% and 50% DC, baseline and response duration are equal to tone burst duration for each cycle. The membrane potential variation ΔV was defined as the difference between the membrane potential median value during stimulus onset (r1 in the figure) and its median value during the preceding baseline (b1 in the figure). (b) Violin plot [37] of baseline variation, defined as the difference between membrane potential pre and post stimulus onset (median Bi+1- median Bi), for each pressure amplitude and DC (95 ms at DC 5%, 50 ms at DC 50%, 300 ms at DC 100%). Asterisks indicate post-hoc significant inter-DC differences (p < 0.05). (c) Membrane voltage variation over the three cycles at DC 5% and 50% for pressure 12 kPa. (d) Membrane potential variation for each pressure amplitude and DC. (e) Median membrane voltage variation for each stimulation protocol setting. (f) Stimulation intensity for each experimental protocol. g) Median membrane potential response as a function of the intensity. Color code in (f) and (g) indicates intensity binning: Ispta is binned into five groups: very low ≤ 0.5 mW/cm^2^ < low ≤ 2.5 mW/cm^2^ < medium ≤ 5 mW/cm^2^ < high ≤ 10 mW/cm^2^ <very high, with respectively 101, 110, 108, 97, 73 recorded traces.

We delivered low-intensity, 490 kHz US in blocks of 3 stimuli of 100 ms each, with a DC of 5, 50 or 100% (continuous stimulation, all reported in Fig. 1(e)) and a root mean square pressure (P_rms_) of 8, 12, 16 or 20 kPa (see Materials and Methods for details). The spatial mapping of the pressure within the acoustic field was measured using a hydrophone and the P_rms_ was evaluated in the Petri dish containing the pinned ganglia (Fig. S2 and Fig. S3). The response and baseline intervals were defined respectively as the on-state of the stimulation (tone burst duration) and as the interval of the same duration preceding the stimulus onset (Fig. 2(a)). Note that the US-induced depolarization outlasted the tone burst duration for pulsed protocols and the duration of continuous stimulation (Fig. 2(a)). Post- and pre- stimulus membrane voltages were compared (B_i_, Fig. 2(a)); increase in the membrane potential associated to the stimulus depended on both pressure and DC, but not on their interaction (two-way ANOVA, p = 0.0272, p < 0.0001, p = 0.32 respectively) (Fig. 2(b)). For EL triggers, membrane potential during the 100 ms post-stimulus time was lower than the pre-stimulation level, in stark contrast to what observed for US (Fig. S1).

We also checked whether the membrane potential response to US stimulation (ΔV), defined as the negative difference between the median membrane potential during baseline and its value during the interval with tone burst stimulation (Fig. 2(a)), was different across the three consecutive stimulation cycles for 5 and 50% DC. We found that there was no significant inter-cycle difference in the response for fixed value of pressures. For instance, for 12 kPa (see Fig. 2(c)) a two-way ANOVA failed to detect a significant difference in the response across the cycle numbers (F = 0.38, p = 0.68) as well as a significant effect of the cycle number x DC interaction (F = 0.17, p = 0.85), while there was a significant difference among the DCs (F = 7.19, p = 0.0082). Similar results were obtained for all pressure levels (results not shown). Consequently, in the following analysis we will always consider the average response amplitude over all cycles.

The relationship between the stimulation features and the peak membrane potential depolarization was then investigated for the various combinations of pressure and DCs. The membrane potential depolarization response ΔV was found to increase with both DC and pressure parameters (Fig. 2(d)–(e)). This is coherent with the fact that during each stimulation the effect of US on the membrane potential is integrated over time (see for instance Fig. 2(a)). A two-way ANOVA with factors DC and P_rms_ detected a significant difference in ΔV across different DCs (p ≪ 0.0001) and pressures (p = 0.0002), as well as for the DC x P_rms_ interaction (p = 0.021) (Fig. 2(b)).

To measure the total acoustic exposure, taking into account the pulsed protocol, the spatial-peak temporal-average Intensity (I_spta_) for each stimulation was then computed as

\begin{equation*}
{I_{spta}} = \frac{{P_{rms}^2}}{{\rho c}}*DC\tag{1}
\end{equation*}

where *ρ* and *c* are approximated to the density and the speed of sound in the water (see the experimental setup in Fig. 1). It was found that membrane depolarization ΔV associated to US grew linearly with I_spta_ (R^2^ = 0.78, p = 0.0001, Fig. 2(g)).

We concluded that the US triggered sub-threshold membrane potential depolarization did not depend simply on the temporal peak stimulation intensity but rather on its integral over time: the effects of the stimulation accumulate linearly. In the following sections, we consider the effect of the stimulations only as a function of I_spta_.

### B. Spiking Activity in Response to US Stimulation

**Figure 3.**
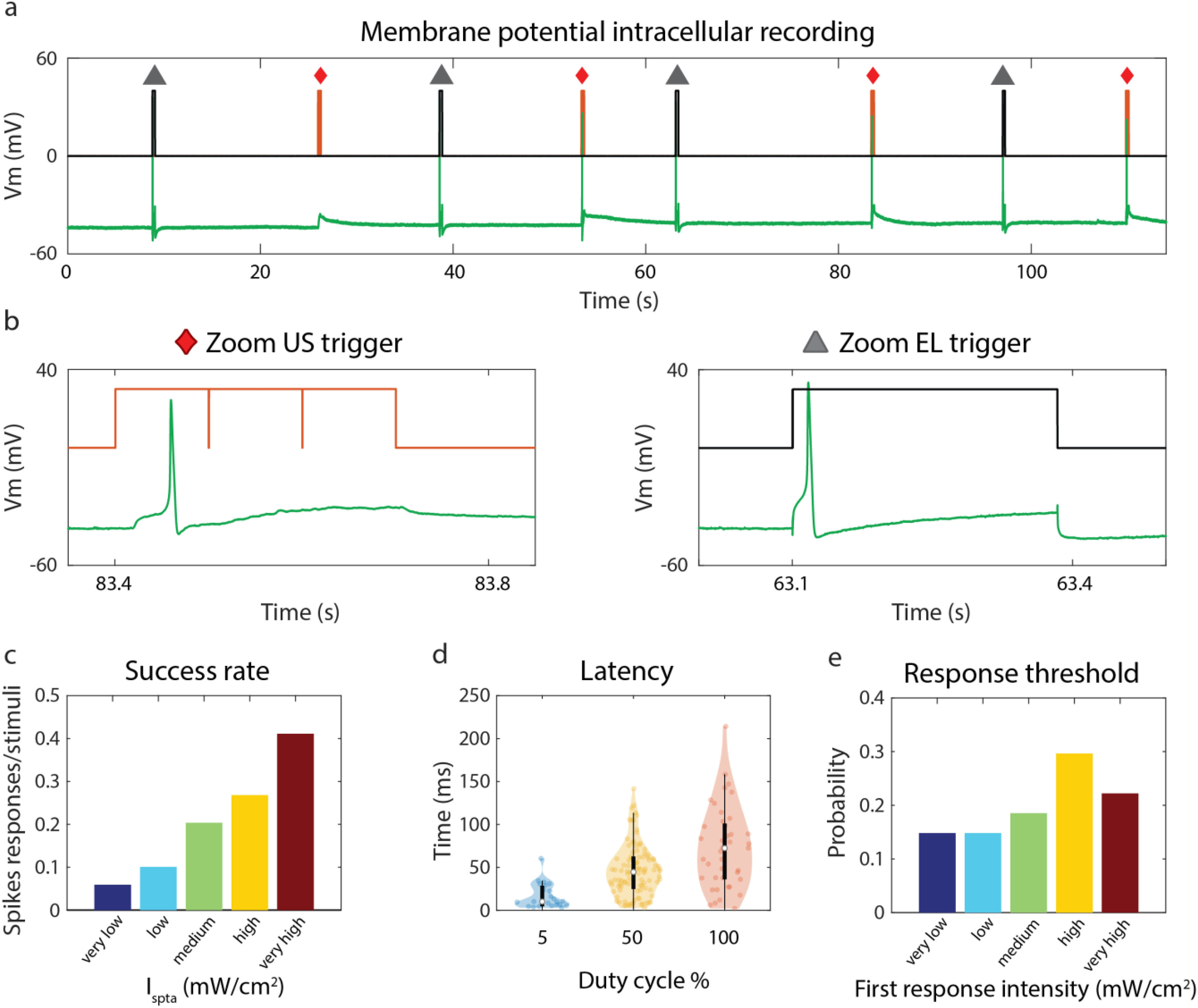
US parameters and firing activity. (a) Example of N cell intracellular membrane potential (green) during US (red diamond) or EL (grey triangle) stimulations. Red line indicates US trigger. Black line indicates EL stimulation trigger. The response to EL stimuli is recorded to compare the spikes characteristics with the US induced and to verify cell health. A time interval of 20–30 s between consecutive stimulations allows the cell to recover. (b) Zoom of the US trigger signal (red and the EL triggered signal (black); recorded membrane potential (green). (c) Success rate as a function of the intensity, binned into 5 groups. The considered spike detection time window for each stimulation lasted 400 ms from the stimulus onset (stimulus duration of 300 ms + 100 ms post stimulation). (d) Distribution of latency as a function of DC of all US-triggered spikes. (e) Distribution across neurons of intensity associated to the first response. Intensity ranges are defined as in Fig. 2(f): very low ≤ 0.5 mW/cm^2^ < low ≤ 2.5 mW/cm^2^ < medium ≤ 5 mW/cm^2^ < high ≤ 10 mW/cm^2^ <very high.

A key experimental result of our work is that US induced spiking activity in 27 over the 44 recorded N cells (see a representative recorded trace in Fig. 3(a)). In order to compare the different spike-triggering mechanisms we alternated US and EL stimulations able to induce firing activity (Fig. 3(b)). Note that the latency was longer for US than for EL stimulation, coherently with our hypothesis of a cumulative effect of US, as if the accumulation of the effect over time required to trigger the action potential determined the latency. Coherently with results in Fig. 2, the success rate (spike elicitation probability) averaged over all neurons depended on both pressure and DC: a two-way ANOVA with factors DC and pressure detected a significant difference between the DCs (F = 6.02, p = 0.003) as well as the pressures (F = 2.84, p = 0.039), but only a tendency toward DC x pressure interactions (F = 6.18, p = 0.082). Indeed, the stimulation success rate was proportional to the intensity (R^2^ = 0.36, p = 0.0442, Fig. 3(c)). Analysis of the spiking activity in the intervals preceding and following the stimulation showed that success rate was always higher than at baseline not only during the stimulation, but also in the following hundreds of ms (Fig. S4), highlighting the presence of a long-lasting effect of US on spiking activity. Spiking activity during stimulation steadily increased with stimulation intensity (R^2^ = 0.96, p < 0.01) and a similar, although not significant, trend was observed for post-stimulation activity (R^2^ = 0.59, p = 0.09).

Another important result came from the latency analysis, measured as the time delay from the tone burst onset to the AP peak. We found that the median of latency distributions increased with the DC (Fig. 3(d)), and the most likely value was close to the duration of the tone burst in case of 5% and 50% DC. This is coherent with the fact that the membrane potential grows monotonically during US stimulation, and consequently the highest probability of firing is at the end of the stimulation. Indeed, a shorter duty cycle was associated to a smaller fraction of responding neurons (Fig. 3(c)) due to the smaller depolarization associated to the lesser cumulative effect. In other words, when we increased the duty cycle we triggered a far larger number of cells, but all the cells that responded only with the longer duty cycle will respond at the end of the duty cycle, hence shifting the latency distribution toward larger values. In case of 100% DC the latency was always shorter than stimulation duration, probably because a saturation effect was reached. These results again indicate that the effect of US accumulate over time.

Interestingly, the latency distributions confirmed that a large fraction of spikes was fired after the end of tone bursts indicating that US can induce long lasting effects even tens of ms after its end. This highlights a key difference with EL-triggered spikes, whose peak occurred immediately after current onset as expected (Fig. 3(a), Fig. S1a).

We also computed the minimal intensity required to elicit spiking activity across all neurons. The resulting distribution showed a maximal probability at moderately high intensities (Fig. 3(e)). Several cells indeed exhibited sufferance in the range we labeled as ‘very high’. This suggests that for each cell there might be an intrinsic intensity dependent activation threshold and a higher intensity sufferance threshold; however, there is large variability among tested cells.

We wondered if mechanosensitivity was a sufficient condition to have US-triggered APs. We stimulated then another kind of leech mechanoreceptor, the P cells (n = 23, see Materials and Methods for details), which is physiologically sensitive to lighter stimuli than the N cells. We found that the membrane potential response was linearly correlated with the intensity of the stimulus also for P cells, (R^2^ = 0.74, p = 0.0007, Fig. S5a), but such responses were much smaller than those elicited by the same intensities in N cells (paired t-test p = 0.0001, Fig. S5b). Due to this weak sensitivity of the membrane potential to US, the occurrence of action potentials was a very rare event (n = 6 out of 222 stimulations, Fig. S5c). This shows that, notwithstanding the common function and the presence of mechanosensitive channels in both kind of cells, N cells displayed a significantly stronger sensitivity to US than P cells.

### C. Comparison Between US- and EL-Induced APs

**Figure 4.**
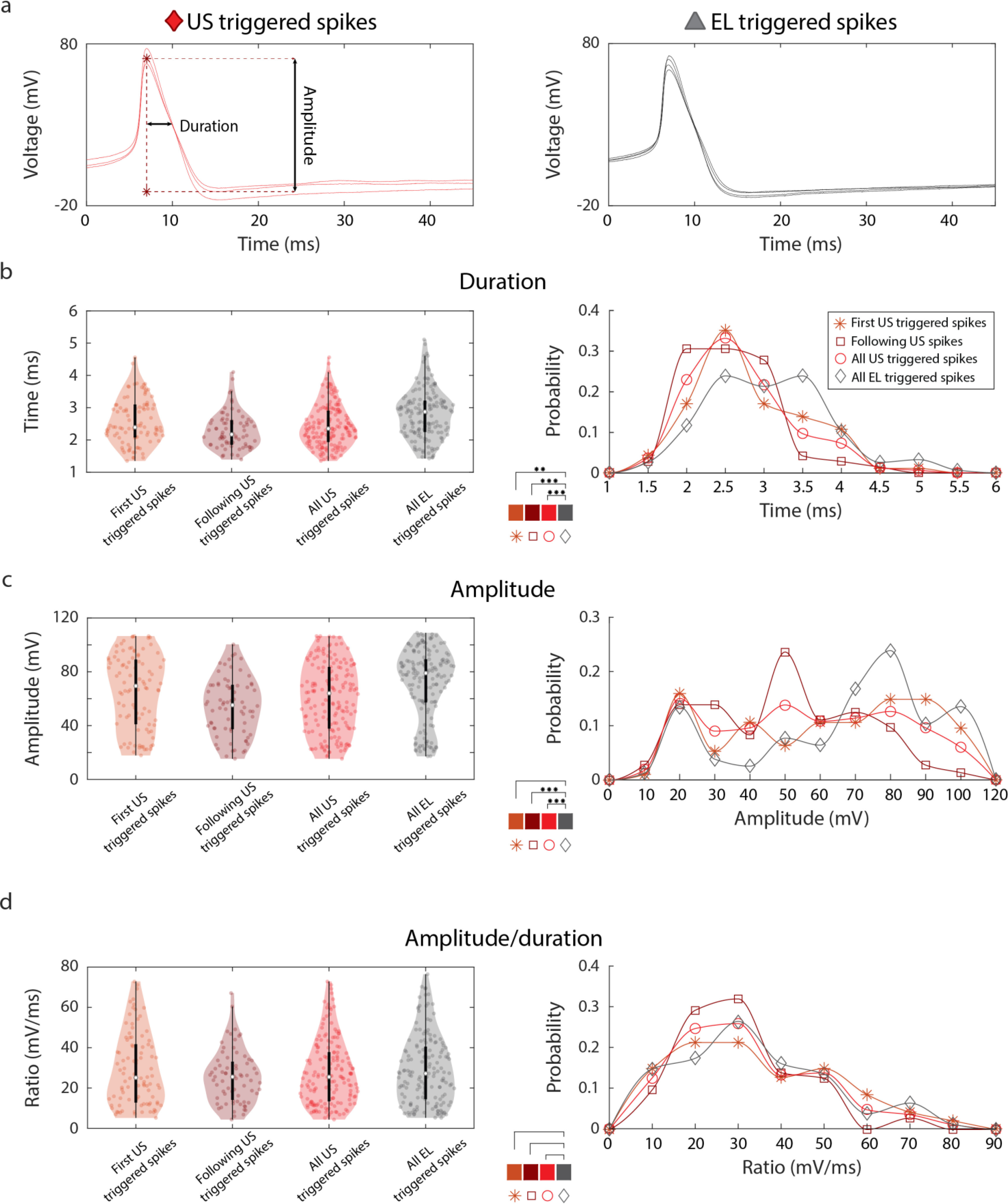
AP features in US and EL stimulation. (a) Shape of APs triggered by US (left 5 spikes) and EL (right 3 spikes) stimulation extracted from the trace in Fig. 3(a). Left US plot shows definitions of amplitude (from peak to subsequent minimum value) and duration of early repolarization (width at half amplitude of declining phase). (b) Left: median, interquartile range and dispersion of first spikes triggered by US (orange), following spikes triggered by USs (brown), the union of the two sets (red), and spike triggered by EL stimulation (grey). Right: comparison between the probability distributions of the durations of the different sets of spikes. Subplot in the center indicates significant differences between distributions. (c) Analysis of the distributions of the amplitudes of the four spike categories defined in (b). (d) Analysis of the distributions of the amplitude/duration ratios of the four spike categories defined in (b).

We established that US is able to modulate membrane potential and trigger APs. To further characterize the specific effect of US, we compared with the same set of stimulations used in the previous section the shape of the APs triggered by US (n = 166 spikes) and EL (n = 155 spikes) stimulations. As previously observed (Fig. 2(a)–(b)), US produced long lasting effects on membrane voltage. We thus focused on fast transient characteristics, i.e., AP amplitude and duration, respectively defined as the voltage difference between AP peak and the subsequent minimum, and the duration of the decaying phase at half amplitude, referred as early re-polarization phase (Fig. 4(a)).

The first APs triggered by US on each stimulus window (n = 94/166 spikes) and the following were analyzed separately to check for possible memory-effects of US; first-triggered APs do not always correspond to first US stimulus (tone burst during stimulus nor first stimulus on recorded trace). The total dataset of US triggered APs showed significantly shorter duration (2.35 ± 0.45 ms) compared to EL triggered APs (2.87 ± 0.7 ms, KW test, p = 6 * 10^−6^) (Fig. 4(b)). We observed that duration of the early repolarization phase of following US triggered APs (2.17 ± 0.4 ms) was shorter than that of first US triggered APs (2.39 ± 0.5 ms, KW test, p = 0.014). Considered separately, durations of both first and following US triggered APs were significantly shorter than those of EL triggered APs, also with statistically significant differences (respectively p = 0.01 and p = 4 * 10^−7^ KW test). Significant differences were also observed when comparing AP amplitudes (Fig. 4(c)). Overall the total dataset of US triggered APs (64 ± 45 mV) showed a significantly smaller amplitude compared to those of EL triggered APs (79 ± 15 mV, p = 1 * 10^−4^). The amplitude of the following US triggered APs (55 ± 16 mV) was lower than that of first US triggered APs (70 ± 47 mV), with a statistically significant difference (KW test p = 0.01). Taken separately, both first and following US triggered APs were significantly smaller than EL triggered APs, although statistically significant difference was only found for the latter comparison (respectively p = 0.14 and p = 4 * 10^−7^). Note that also these differences in AP shape could be associated to the ‘long lasting effect’ of US compared to the EL (see Conclusions).

We further investigated spiking responses to US taking into account not only the dimensions, but also the shape of the APs, comparing the ratio between amplitude and duration of the AP, which is proportional to the slope of the decay phase of the APs (Fig. 4(d)). Interestingly, and coherently with the fact that both amplitude and duration were found to have higher values for EL triggered than US triggered APs, we found that the ratio follows the same trend and belongs to the same distribution. We found no statistically significant difference between the amplitude/duration ratio of EL triggered APs (27.4 ± 12 mV/ms) and US triggered APs (25.6 ± 11.5 mV/ms, KW test, p > 0.5). Moreover, there was no difference between first (25.5 ± 13 mV/ms, KW test, p > 0.5), and following US-triggered spikes (25.7 ± 9 mV/ms, KW test, p = 0.26).

## III. Conclusions

Despite the increasing amount of research on the effects of US stimulation on the nervous system, the biophysical dynamics underlying the generation of AP following US stimulation is still unclear. Several small animal models have been used to test US stimulation thanks to the possibility to perform experiments in a very controlled context. We selected the leech *Hirudo Medicinalis* for our studies as it is a consolidated neurophysiological model suitable for the analysis of such complex mechanisms. Moreover, the isolated leech ganglion preparation (Fig. 1) was found to be suited for performing intracellular recording during US stimulation.

We have shown that US has a direct depolarization effect and elicits spiking activity in leech N neurons. Our work is the first study establishing useful guidelines for US stimulation of excitable cells, showing that the induced activity depends on the applied acoustic I_spta_ (Fig. 2 and Fig. 3).

More in detail, it was demonstrated that the effects of US leading to increased membrane depolarization for higher pressure amplitude and increasing DC can be summarized by the I_spta_ (Fig. 2(f)), and that this linearly modulates sub-threshold depolarization (Fig. 2(g)) and is proportional to spike probability (Fig. 3(c)). Crucially, sonication produced long lasting effects on membrane voltage (Fig. 2(a)–(b)), leading to increased spiking activity outlasting the stimulation (Fig. S5, Fig. 3(d)).

These results also establish well-defined relationships between US driving parameters and ensuing spiking activity, which might be useful in the design of future experiments. In particular, while duty cycle duration and intensity can be independently modulated, all that matters seem to be their product. This can have practical consequences, as choosing to achieve a given response by doubling the intensity (if a fast response is needed) or by doubling the stimulus duration (to avoid damages to the cell).

Finally, US- and EL-triggered spiking activity were compared by considering fast transient characteristics, i.e., the amplitude and early repolarization duration of the AP. Interestingly, we found that US-induced APs differ from EL-induced ones both in amplitude and early repolarization duration, but not in the ratio between these two quantities. The combination of these results suggests that the mechanism inducing spikes in the two cases may involve the same ion channels as the waveform shape is preserved. First US triggered APs amplitude is higher than the following ones, probably as a consequence of the residual depolarization effect produced by the sonication.

Several works suggest that US acts on voltage gated ion channels [4], [13], [31], [32], [35]. However, the exact dynamics of this interaction is still unclear. Leech N neurons exhibit standard AP dynamics, with a Na^+^-driven depolarization phase followed by a slow K^+^-driven repolarization phase [30]. The fact that US-triggered APs show qualitatively similar waveforms to those of EL-triggered APs (Fig. 3(b)) with quantitatively similar repolarization rates (Fig. 4(d)), likely indicates that upon sonication, both Na^+^ and K^+^ channels conserve standard kinetics. It is thus unlikely that US exerts an indistinct, long-lasting action on these ion channels, as that would rather drive membrane voltage towards a stable plateau potential. Hence, a distinct ion channel population likely mediates our observation of US-triggered sub-threshold depolarization (Fig. 2). As this depolarization linearly depends on the stimulus intensity but not on its specific temporal pattern of application, we hypothesize that the affected channels exhibit a rather slow temporal kinetics, such as that of mechanosensitive channels that are natively expressed in leech N cells. The hypothesis that US can regulate the activity of mechanosensitive ion channels was previously proposed [13]. Moreover, the possible long-lasting effect of US on such channels could likely explain the observation of an accumulative depolarizing effect during second and third US stimuli, resulting in a lower amplitude of the APs. The fact that more than 50% of the recorded cells responded with spikes to US suggests a threshold mechanism associated to US stimulus intensity. These results are in accordance with *in vitro* studies [31], [32], which observed in different regions of rat hippocampal slice cultures that US induced intensity dependent responses, and hypothesized a threshold mechanism and a fatigue effect associated to US stimulus intensity. Moreover, the high variability of responses and success rates observed across recorded cells, similarly as in previous studies [8], [11], [31], [32], could be explained by different densities of mechanosensitive ion channels expressed in the same cell type. Possible future experiments addressing the identification of the channels affected by US could include patch clamp recordings, gene protein expression and channel silencing.

More specifically, a possible explanation to the different sensitivity to US of P and N cells could lie on the recent finding of frequency specificity of the classes of mechanosensory neurons in the leech [33]. In this work the authors find that N cells are effectively low-pass filtering voltage oscillations while P cells act as high pass filters. Therefore, as US-induced membrane potential deflections have slow time scales even when the pulses are short lived (Fig. 2(a)), the P cells are not sensitive to this stimulation. Moreover, the integration of the depolarizing effect of the US reported in the subthreshold responses of our paper is in line with a low-pass filter voltage membrane behavior. This finding shows that the temporal scale of the stimulation could also have a strong effect even when the cells show an excellent mechanosensitivity and opens the possibility to selectively modulate different mechanoreceptors according to their specific frequency sensitivity.

The parameter set tested in the present work is limited to three DCs (5-50 and 100%) and four low pressure levels (8-12-16-20 kPa), in accordance with ranges considered safe for human US imaging [34]. Stimulation center frequency has been set to 490 kHz in accordance with previous studies [9], [13]. Further studies are needed to observe the effect of different stimulation protocols. It is in fact likely that the inhibition and excitation effects could have different thresholds, as the stimulation could be more effective on different types of channels, or membrane proteins, which coexist in the same cell.

We achieved so far only a neurophysiological characterization of the responses associated to US in a specific kind of cells, not only providing another proof that US neuronal activity modulation is possible, but also assessing some operational rules that might apply also to other neuronal populations. We are currently conducting studies on other animal preparations to assess the generality of our research and investigate more deeply ion channel dynamics upon US to further understand the working principle that stands behind US neurostimulation.

This work lays the ground for future studies on ultrasonic stimulation and possibly their use in non-invasive neuroengineering biomedical applications.

## IV. Materials and Methods

### A. Animals and Preparation

Leeches (*Hirudo medicinalis*) were purchased from Ricarimpex (Eysines, France) and kept at 5°C in tap water dechlorinated by aeration for 24 h. They were dissected in chilled Ringer's solution with the following composition (mM): 115.0 NaCl, 1.8 CaCl_2_, 4.0 KCl, 12.0 glucose, 10 Tris maleate, buffered to pH 7.4 with NaOH. A longitudinal incision was performed on the dorso-medial side of the animal to expose the chain of ganglia. Surrounding tissues, including the ventral main blood vessel, were carefully removed without touching the nervous tissues (Fig. 1(b)). In parallel, a custom recipient was made by removing via laser cut the plastic bottom of a Petri dish and replacing it with a 25 μm thick US transparent polystyrene membrane (Goodfellow, Huntington, Cambridge, UK), subsequently coated with PDMS (ratio of monomer:curing agent = 5:1); the membrane was cut and removed from the bottom after PDMS polymerization. Finally, a ventral ganglion was extracted, fixed ventral side up on the custom recipient via metal pins located on the roots and connectives, and kept in fresh Ringer's solution (Fig. 1(a)–(c)).

Previous attempts to measure membrane voltage with current clamp recordings evidenced the ability of US to elicit action potentials on CA1 pyramidal neurons [4], and on Xenopus oocytes [13], but the cell seal during sonication was not stable. The authors postulated then that the resonance of the intracellular electrode was responsible for the ineffectiveness of the experiments at low US frequency. Here we observed instead that electrode instability and subsequent cell leakage could also be caused by the induced relative movement of the cultured cells/oocytes (poor adhesion and fluctuation in the medium) with respect to the substrate and the glass capillaries, originating during US. To overcome this limitation, we applied several counter-measures detailed below. To further verify that cell seal was maintained during US sessions, EL stimuli were applied and cell health was monitored; we observed that the EL response in US-irresponsive cells was not affected by the US stimulus and was preserved after the whole protocol execution. To ensure stable cell seal, we secured the micromanipulator for electrophysiology recordings on a rigid support and decided to record from the intact ganglion stretched and secured through metal pins on a thin PDMS substrate. In order to reduce substrate vibrations due to sonication, we increased the crosslinker concentration from 10:1 to 5:1, obtaining a good stability of the sample during the experimental sessions, as no vibration artifacts were observed on the recorded traces.

### B. Intracellular Recordings

Nociceptive (N) cells in the isolated ganglion were impaled by a sharp glass capillary filled with 3 M potassium chloride, containing an Ag/AgCl electrode (input resistance ≈ 10 MΩ) to record intracellular potential and deliver electrical pulses (Fig. 1(a), inset). The ground connection was placed at the border of the Petri dish, immersed in the Ringer's solution. Recorded signals were amplified with Axoclamp-2b amplifiers (Axon Instruments, Foster City, CA, USA), digitized, stored in a personal computer and analyzed with the pCLAMP8 software (Axon Instruments, Foster City, CA, USA). Nociceptive (N) cells and pressure (P) cells of leech ganglion were identified in different sessions under optical microscope and impaled. A total of 47 N and 23 P cells were employed in this study.

### C. Ultrasound and Electrical Stimulation

US was applied on leech ganglion by using a 44 mm diameter PZT (Lead zirconate titanate) unfocused transducer (Precision Acoustics LTD, Dorchester, UK) immersed in a polymethyl methacrylate (PMMA) tank filled with degassed deionized water, atop of which the recipient containing the ganglion was placed. The transducer was driven by a waveform generator (Agilent33220A Keysight Technologies, Santa Rosa, CA, USA) in series with a 50 dB gain radio frequency power amplifier (240L, Electronics & Innovation, Rochester, NY, USA). The US beam reached the ganglion (placed at a distance of 165 mm from the surface of the transducer) from the dorsal side. Note that the whole ganglion was within the ultrasound field. Sinusoidal tone bursts at 490 kHz, with pressure amplitude from 8 to 20 kPa were delivered at a pulse repetition frequency (PRF = 1/PRP) of 10 Hz, for a total stimulus duration of 300 ms. The duty cycle (DC) was fixed at 5%, 50%, 100% (Fig. 1(e)), therefore the spatial-peak temporal-average intensity (I_spta_) varied from 0.2 to 27 mW/cm^2^ (Fig. 2(f)). According to our estimates the displacement of the whole ganglion due to ultrasound waves should be at most 6 μm [35].

Each recording session consisted at least of three identical US windows of 300 ms, and electric pulses were used to generate a single spike in N cells prior and after each US. The US protocol was interrupted if no response to electrical stimuli was observed. The considered spike detection time window for each stimulation lasted 400 ms from the stimulus onset (stimulus duration of 300 ms + 100 ms post stimulation). Electrical stimuli were manually provided with a variable duration (ranging from 0.25 to 1 s) and amplitude (current ranging from 1 to 5 nA).

### D. Ultrasound Calibration

The US transducer was characterized in free field conditions both in terms of US pressure field mapping and intensity vs. driven voltage calibration.

The acoustic field was mapped by measuring the generated pressure with a 2 mm PVDF needle hydrophone (Precision Acoustics, Dorchester, Dorset, UK) at different locations, using a three-axis step-by-step motorized positioning frame (XYZ BiSlide, Velmex, Bloomfield, NY, USA). A dedicated LabVIEW program (National Instruments, Austin, TX, USA) allowed synchronization between the wave generator, motors and signal acquisition from an oscilloscope (7034 B, InfiniiVision, Agilent Technologies).

Additionally, the root mean square pressure (P_rms_) and the spatial peak pulse average intensity (I_sppa_ = P_rms_^2^/(ρ c)) were evaluated at the experimental distance of 165 mm at different driving voltages, where ρ and c are approximated to the density and speed of sound of water, respectively. The driven voltage was measured at the output of the power amplifier. Spatial peak temporal average Intensity (I_spta_) was easily derived by multiplying the I_sppa_ and the duty cycle (DC) used in the stimulation protocol.

Finally, in order to consider possible acoustic reflection and attenuation phenomena due to the experiment setup configuration, additional intensity measurements were performed by positioning the hydrophone tip inside the custom recipient used during experiments. Free field and the experimental setup measurements are reported in Fig. S2; transducer characterization results are reported in Fig. S3.

### E. Data Analysis and Statistics

The recorded traces were analyzed with MATLAB R2017b (The MathWorks, Inc., Natick, MA, USA). No band-pass filtering was applied to the measured intracellular potential to keep the original waveform for each cell; the considered signal window was detrended (1st order polynomial function) before the peak analysis. Amplitude and early repolarization duration of the spikes were measured: the amplitude is defined as the difference between the peak voltage value and the subsequent minimum; the early repolarization duration is measured at half prominence in the drop phase of the spike, to avoid the possible artifact on membrane potential due to the stimulation start, which is particularly effective on low amplitude spikes. The threshold for spikes detection was set to 15 mV above baseline; subthreshold spikes were also analyzed.

Statistical significance in spike characteristics differences between US and electrically triggered spikes and subthreshold spike analysis (membrane voltage variation between the three stimulation bursts and the difference for each input pressure among duty cycles) was evaluated using the Kruskal-Wallis one-way ANOVA test. A p-value of 0.05 indicated a significant difference in the analyzed distributions; datasets were plotted with the violin plot method [36], which allows for the visualization of the full distribution of the dataset. Response latency, defined as the time difference between the US stimulus start and the subsequent spike peak event, was also analyzed.

## Supplementary Materials

Supplementary materials display five figures. Fig. S1 (related to Fig. 2) concerns the estimation of observation time and membrane potential baseline variations for EL stimulation. Fig. S2 and Fig. S3 (related to Fig. 1) concern US calibration setup and results. Fig. S4 (related to Fig. 3(c)) focuses on the analysis of US-APs success ate as a function of acoustic intensity and stimulus onset and offset. Fig. S5 (related to Fig. 2 and Fig. 3) concerns the comparison between P and N cells in response to ultrasonic stimulation.


