# Supplementary material for: Ultrasound Stimulations Induce Prolonged Depolarization and Fast Action Potentials in Leech Neurons
Source: IEEE Open J Eng Med Biol. 2020 Feb 14;1:23–32. doi: 10.1109/OJEMB.2019.2963474 (PMC8979621; doi:10.1109/OJEMB.2019.2963474)
Supplement: Supplementary file 1 [file supp1-2963474.pdf]

Supplementary Materials

Ultrasound stimulations induce prolonged depolarization and fast action potentials in leech neurons

Francesca Dedola, Francesco Paolo Ulloa Severino, Nicolò Meneghetti, Théo Lemaire, Andrea Cafarelli, Leonardo Ricotti, Arianna Menciassi, Annarita Cutrone, Alberto Mazzoni, Silvestro Micera

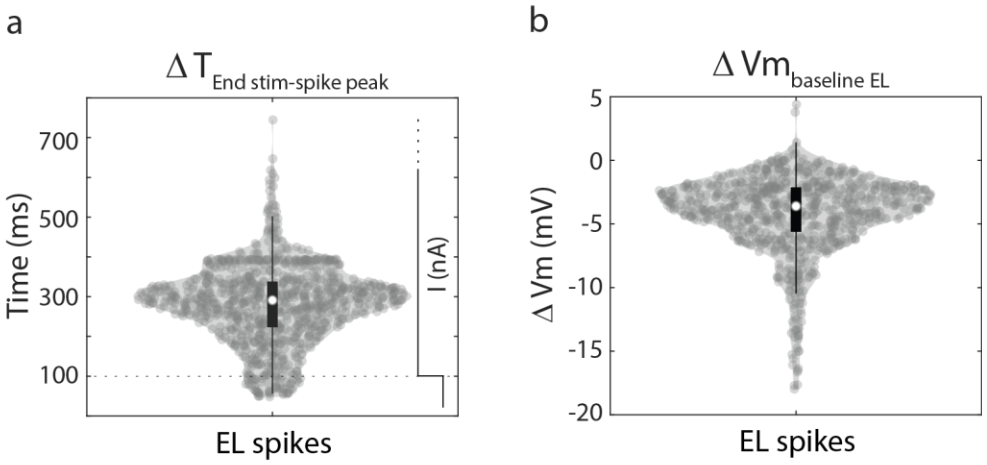

Fig. S1 (related to Fig. 2). Estimation of observation time and membrane potential baseline variations. a) Analysis of the EL stimulations to estimate the probability of spike detection after the end of the stimulus; only few cells showed spikes during the considered post stimulation time interval (100 ms). The dotted line corresponds to the end of EL trigger. Time measured from 100 ms after trigger end to preceding spike peak. b) Baseline variation on EL stimulation, defined as difference between membrane potential post and pre-current onset. A time window of 100 ms is considered for post and pre-stimulation baseline analysis. Membrane voltage after EL stimulation is lower on the considered observation window.

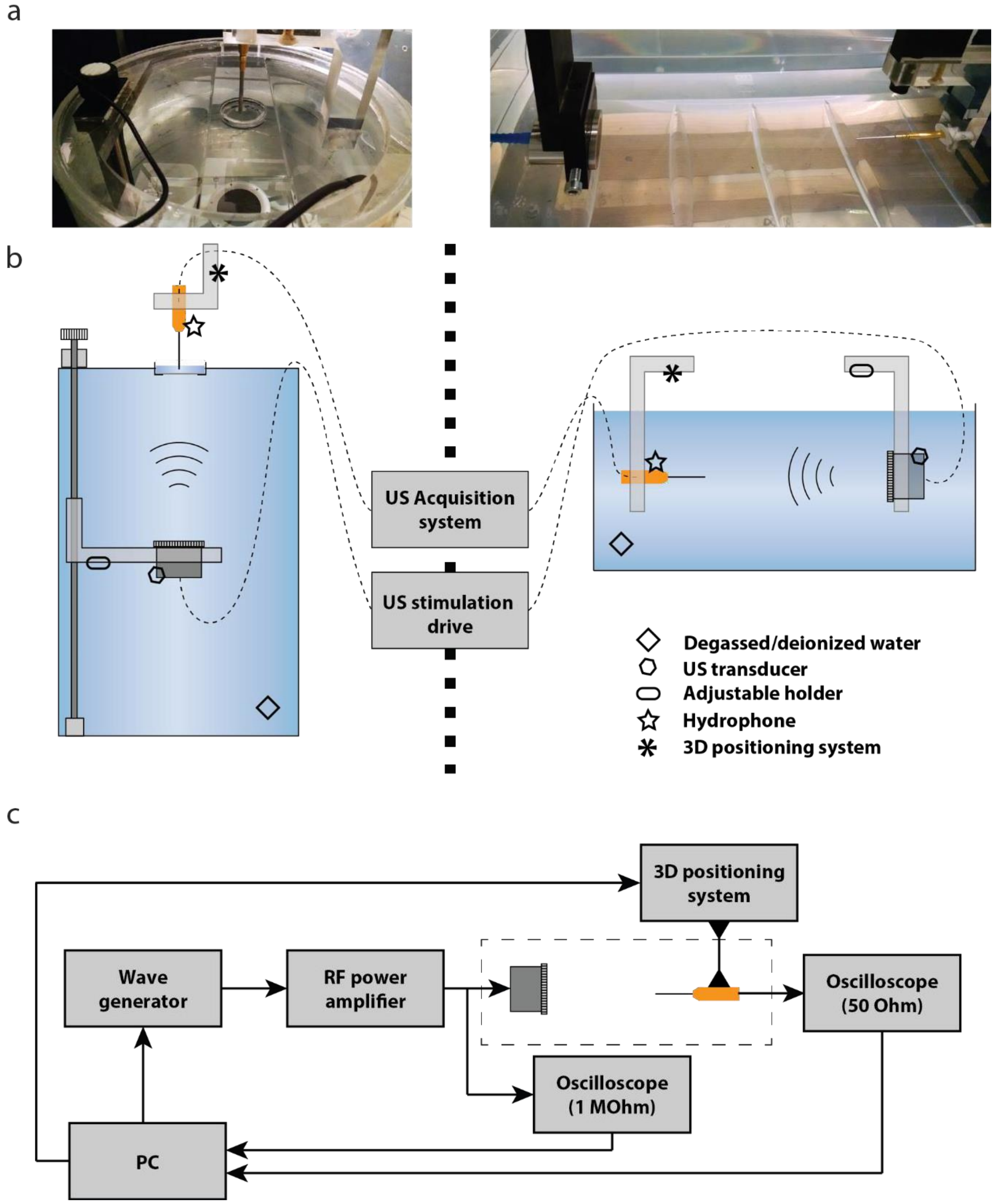

Fig. S2 (related to Fig. 1). US calibration setup a) left: calibration into the experimental setup; right: free field calibration setup. b) Scheme of the calibration measurement system. c) Detailed block representation of US/acquisition setup.

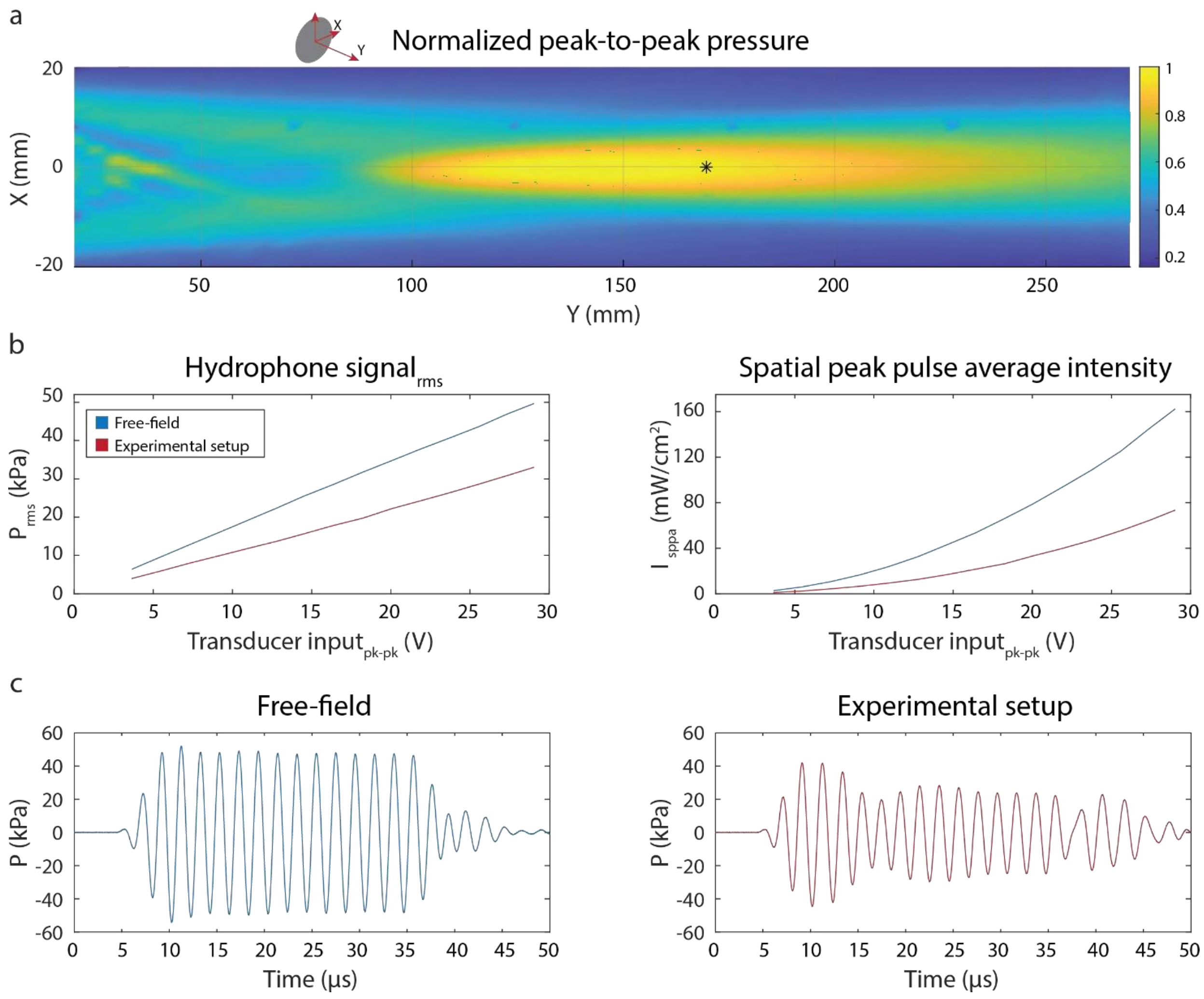

Fig. S3 (related to Fig. 1). US calibration results. a) 2D spatial mapping of normalized peak-to-peak pressure produced by the transducer in free field. Transducer focus is at 140 mm. Asterisk indicates the distance between the transducer and target employed during experiments, i.e. 165 mm. b) On left, hydrophone root-mean-square pressure (P_{rms}) signal vs driving voltage; low pressure calibration of the transducer shows that pressure is linear with the input voltage. The signal measured in the experimental setup is attenuated with respect to the free field condition. On right, calculation of spatial-peak pulse-average intensity, I_{sppa}; free field (blue), experimental setup (red). The measure has been conducted at 165 mm from transducer surface. c) Example of time variation of the pressure signal in free-field (blue) and experimental setup (red). It can be observed that the second is attenuated and a post stimulus onset effect is introduced by the experimental setup.

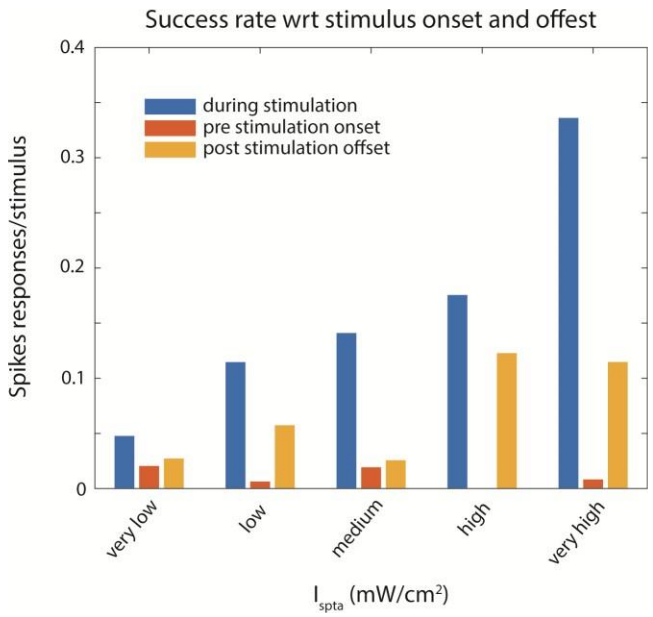

Fig. S4 (related to Fig. 3c). Action potential success rate as a function of acoustic intensity (binned into 5 groups) and of US stimulus onset and offset. Blue: AP success rate during US stimulus (i.e., during a time window of 300ms following US stimulus onset). Red: AP success rate during a window of length equal to the stimulus duration (i.e., 300 ms) but preceding the stimulus onset. Yellow: AP success rate during a window of length equal to the stimulus duration (i.e., 300 ms) but following the stimulus offset.

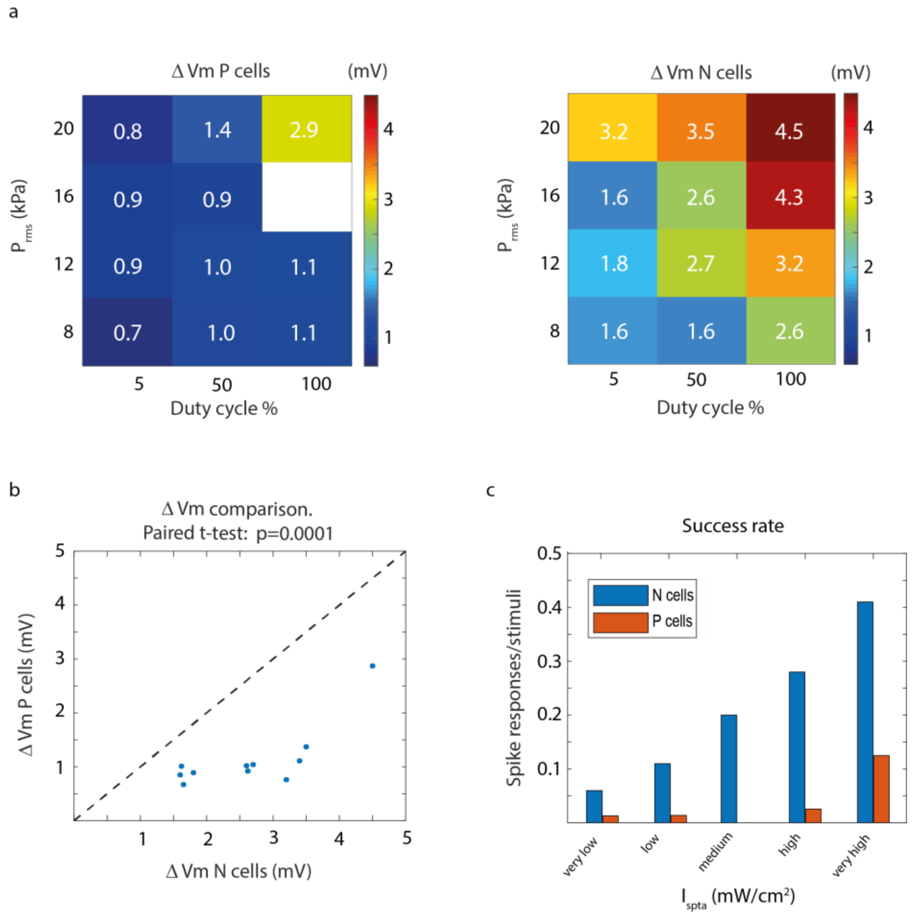

Fig. S5 (related to Fig. 2 and Fig. 3). Comparison between responses to US of P and N mechanoreceptors. a) Left: Same as Figure 2e for P cells stimulation. Empty square indicates that no cell responded to that particular parameters combination. Right: Figure 2e rescaled for clearer comparison. b) Comparison of average membrane potential deflection for P and N cells across stimulation intensities. c) Same as Figure 3c, displaying spike responses from P and N cells.
